# Identification of attractive odorants released by preferred bacterial food found in the natural habitats of *C*. *elegans*

**DOI:** 10.1371/journal.pone.0201158

**Published:** 2018-07-23

**Authors:** Soleil E. Worthy, Lillian Haynes, Melissa Chambers, Danika Bethune, Emily Kan, Kevin Chung, Ryan Ota, Charles J. Taylor, Elizabeth E. Glater

**Affiliations:** 1 Department of Chemistry, Pomona College, Claremont, California, United States of America; 2 Department of Biology, Harvey Mudd College, Claremont, California, United States of America; 3 Department of Neuroscience, Pomona College, Claremont, California, United States of America; Duke University, UNITED STATES

## Abstract

Food choice is critical for survival because organisms must choose food that is edible and nutritious and avoid pathogenic food. Many organisms, including the nematode *C*. *elegans*, use olfaction to detect and distinguish among food sources. *C*. *elegans* exhibits innate preferences for the odors of different bacterial species. However, little is known about the preferences of *C*. *elegans* for bacterial strains isolated from their natural environment as well as the attractive volatile compounds released by preferred natural bacteria isolates. We tested food odor preferences of *C*. *elegans* for non-pathogenic bacteria found in their natural habitats. We found that *C*. *elegans* showed a preference for the odor of six of the eight tested bacterial isolates over its standard food source, *E*. *coli* HB101. Using solid-phase microextraction and gas chromatography coupled with mass spectrometry, we found that four of six attractive bacterial isolates (*Alcaligenes sp*. JUb4, *Providenica sp*. JUb5, *Providencia sp*. JUb39, and *Flavobacteria sp*. JUb43) released isoamyl alcohol, a well-studied *C*. *elegans* attractant, while both non-attractive isolates (*Raoultella sp*. JUb38 and *Acinetobacter sp*. JUb68) released very low or non-detectable amounts of isoamyl alcohol. In conclusion, we find that isoamyl alcohol is likely an ethologically relevant odor that is released by some attractive bacterial isolates in the natural environment of *C*. *elegans*.

## Introduction

All organisms must find and detect good food sources that promote growth and survival and avoid detrimental food sources that slow growth or cause illness. For many organisms, including humans, olfaction is a major way of discriminating among different food types [1]. The nematode *C*. *elegans* lives in microbe-rich habitats of rotting fruit and plant matter where they mainly feed on bacteria and likely use olfaction to find bacterial food [2].

It has long been known that *C*. *elegans* is attracted to many volatile compounds that are released by bacteria, including alcohols, ketones, aldehydes, esters and amines, and can discriminate among mixtures of volatile compounds released by different kinds of bacteria [3–6]. Recently, specific attractive volatiles important for attraction to particular bacteria have been identified [7–10]. However, many of these bacteria have been pathogens which likely attract the *C*. *elegans* host to facilitate ingestion and infection [5,11,12]. What volatile chemicals attract *C*. *elegans* to non-pathogenic bacterial food? Diacetyl has been identified as an attractive volatile compound mediating attraction to one non-pathogenic bacterium, *Lactobacillus sp*. that was isolated from rotten fruit where *C*. *elegans* was also found [9].

However, volatile chemicals that attract *C*. *elegans* to additional non-pathogenic strains isolated from their natural environment have not yet been identified. Until recently, little was known about the natural ecology of *C*. *elegans* and its likely bacterial food choices in its natural habitat [2]. Samuel and colleagues cultured bacterial isolates from the same environment that *C*. *elegans* was also co-located [13]. We used bacterial isolates from this collection to ask three questions. First, what natural bacterial isolates does *C*. *elegans* prefer? Second, what natural bacterial isolates does *C*. *elegans* prefer based on only olfactory cues? Third, what are the volatile chemicals released by these attractive bacteria? We found that *C*. *elegans* showed a strong or moderate preference for the odor of six of the eight tested bacterial isolates over *E*. *coli* HB101. We then used solid-phase microextraction and gas chromatography coupled with mass spectrometry (SPME GC-MS) to identify the major volatile chemicals released by the bacterial isolates and found that four of the six attractive isolates (*Alcaligenes sp*. JUb4, *Providenicia sp*. JUb5, *Providencia sp*. JUb39, and *Flavobacterium sp*. JUb43) released isoamyl alcohol while two non-attractive isolates (*Raoultella sp*. JUb38 and *Acinetobacter sp*. JUb68) released very low or non-detectable amounts of isoamyl alcohol.

## Materials and methods

### *C*. *elegans* and bacterial strains

*C*. *elegans* were grown and maintained under standard conditions at 20 °C on Nematode Growth Media (NGM) [14]. N2 worms were grown on NGM plates seeded with *E*. *coli* HB101 ATCC 33694 obtained from the American Type Culture Collection. Bacterial strains (kindly given by Marie-Anne Felix, Institute of Biology of the Ecole Normale Supérieure, Paris, France) were *Alcaligenes sp*. JUb4, *Providenica sp*. JUb5, *Psuedomonas sp*. JUb12, *Raoultella* JUb38, *Providencia sp*. JUb39, *Flavobacterium sp*. JUb43, *Acinetobacter sp*. JUb68, and *Pseudomonas sp*. JUb93 [13].

### Bacterial choice assay

The two-choice bacterial choice assay was conducted as described [11]. Briefly, bacteria were grown overnight in Luria-Bertani media (LB) at 26 °C, centrifuged at 3000 rpm for 5 minutes, and then resuspended in 500 μl LB. The OD600 of a 1:100 dilution of this bacterial suspension was measured. Based on this measurement, the bacterial suspension was diluted with LB to OD600 = 10. 25 μl of each bacterial suspension was spotted onto NGM plates, plates were covered with petri dish lids, and incubated for 5 h at 20 °C. Adult animals were washed three times in S-basal buffer, 50–200 animals were placed near the center of an NGM plate, equidistant from the two bacterial patches, and plate was covered with petri dish lid. Animals were allowed to move freely for 1 hour, then 5 μl of 1 M sodium azide was added to each bacterial patch to immobilize worms on bacterial patches, and then worms were counted. Bacterial choice index equals number of worms on natural bacterial isolate minus number of worms on *E*. *coli* HB101 divided by number of worms on natural bacterial isolate and number of worms on *E*. *coli* HB101. Assays for each condition were repeated at least six times on at least two different days.

### Bacterial odor choice assay

The bacterial odor choice assay is a modified version of the bacterial choice assay and measures olfactory preference for bacteria based only on volatile chemical cues released by the bacteria (adapted from Choi *et al*., 2016). 25 μl of each bacterial suspension (OD600 = 10) was spotted onto NGM plates, plates were covered with petri dish lids, and incubated for 5 h at 20 °C. Then each agar square containing 25 μl bacteria patch was extracted using a metal spatula that was sterilized by placing in ethanol, flaming, and allowing to cool at room temperature briefly before use. An NGM agar square with natural bacterial isolate and an NGM agar square with *E*. *coli* HB101were placed on opposite sides of a petri dish lid. 1 μl of 1 M sodium azide was pipetted on NGM agar directly below bacterial patch on lid in order to immobilize worms that reach the area of the NGM agar plate below the bacterial patch. Immediately after bacterial odor choice plates were prepared, adult animals were washed three times in S-basal buffer, 50–200 animals were placed near the center of an NGM plate, and plate was covered with petri dish lid with bacterial patches. Animals were allowed to move freely for 1 hour and then were counted. Bacterial odor choice index equals number of worms on agar plate under natural bacterial isolate minus number of worms under *E*. *coli* divided by number of worms under natural bacterial isolate and number of worms under *E*. *coli*. Worms were counted as under a bacterial patch if they were within 2.5 cm radius of 1 μl of 1 M sodium azide spot which was placed on agar directly below each bacterial patch. Assays for each condition were repeated at least six times on at least two different days.

### Chemotaxis assays

Chemotaxis assays were performed using 10 cm square chemotaxis plates as described [15]. In brief, assay agar was 2% agar, 1 mM MgSO_4_, 1 mM CaCl_2_, 5 mM phosphate buffer [pH 6.0]. Chemical dilutions were in ethanol at the concentrations indicated in figure legends. 2 μl of diluted chemical were pipetted on one side of the plate, 2 μl of ethanol on the other side, and 2 μl of 1 M sodium azide on both sides to anaesthetize animals that reached odor or ethanol sources. Adult animals were washed two times in S-basal buffer and one time in water, 50–200 animals were placed at the center of chemotaxis plate, plate was covered with lid, and the distribution of animals counted after 1 hour. The chemotaxis index is the number of worms on odor side minus the number of worms on diluent side divided by the total number of worms that have left the origin. Assays for each condition were repeated at least six times on at least two different days.

### Solid-phase microextraction gas chromatography–mass spectrometry

Bacteria were prepared for GC-MS analysis in a similar method as described [10]. Bacteria were prepared in the same manner as for bacterial choice assay. Bacteria were grown overnight in LB at 26 °C, centrifuged, and then resuspended at an OD600 = 10. For each bacterial sample, two NGM plates were prepared, each with 9 spots of 25 μl of bacterial suspension (or LB media) and incubated for 1 h at 20 C. Then 18 squares (each approximately 8 mm × 8 mm) of the NGM agar with 25 μl bacteria suspension or LB media were placed in a GC-MS glass vial at 20 °C for 5 h. Each agar square was extracted using a metal spatula that was sterilized by placing in ethanol, flaming, and allowing to cool at room temperature briefly before use. Samples were collected and analyzed by SPME-GC-MS. Headspace volatile organic compounds (VOCs) were collected for analysis using a DVB/CAR/PDMS SPME fiber for 20 minutes while samples were incubated in a 30 °C water bath. Samples were analyzed using the Agilent 6890 GC System equipped with a Restek, Rtx-5 column and Agilent 5973 Mass Selective Detector. The temperature program was: hold 2 min at 35 °C, increased to 140 °C at a rate of 10 °C /min, and then increased to 250 °C at a rate of 100 °C /min and hold at 250 °C for 3 minutes. MS ranged from 30 to 550 in full scan mode. VOCs were identified with the NIST 11 (National Institute of Standards and Technology) mass spectral library and confirmed with pure chemical standards run following the same parameters as for bacterial samples. Bacterial samples were prepared two or three times on different days and then analyzed and LB control samples were run immediately before or after each bacterial sample. To determine approximate absolute quantities of isoamyl alcohol, an isoamyl alcohol standard of known quantity was run and the quantities of compounds in bacterial headspace were calculated relative to the total peak area of the standard. All standard chemicals were at least 98% purity and were purchased from Sigma-Aldrich (USA).

### Statistical analysis

Means represent data pooled from assays run on at least two different days ≥ 6 replicates. Error bars in all figures are standard error of means. The data were analyzed using statistics described in figure legends with GraphPad Prism v 5.0a for Mac (GraphPad Software, San Diego California USA).

## Results

### Food preference behavior of *C*. *elegans* for natural bacteria

We set out to determine the food preferences of *C*. *elegans* for different bacteria found in their natural environment, to determine the olfactory preferences for odors of these bacteria based only on volatile cues, and to identify the bacterial odorants that were attractive. In particular, in this study, we chose to focus on bacterial strains that are non-pathogenic or mildly pathogenic to *C*. *elegans* because previous studies have identified attractive volatile odors released by pathogenic bacteria [7,10,16]. We wanted to complement these studies by examining attractive odors released by non-pathogenic bacteria. We were fortunate to be able to work with bacterial isolates that had been cultured from natural habitats, including decaying fruit, plant material and invertebrate organisms, where *C*. *elegans* was also found [13].

As this is a large collection with over 200 isolates, we decided to focus on a subset of these bacteria based on the following criteria. First, we chose to examine bacteria belonging to the bacterial phyla *Proteobacteria* and *Bacteroides* because these phyla were among the dominant phyla in this collection [13]. Second, we chose not to work with the two other dominant phyla, *Firmicutes* and *Actinobacteria*, because these bacteria are often sporulating and may lead to laboratory contamination [17,18]. Third, we selected bacterial species that were not known pathogens of *C*. *elegans*.

**Fig 1:**
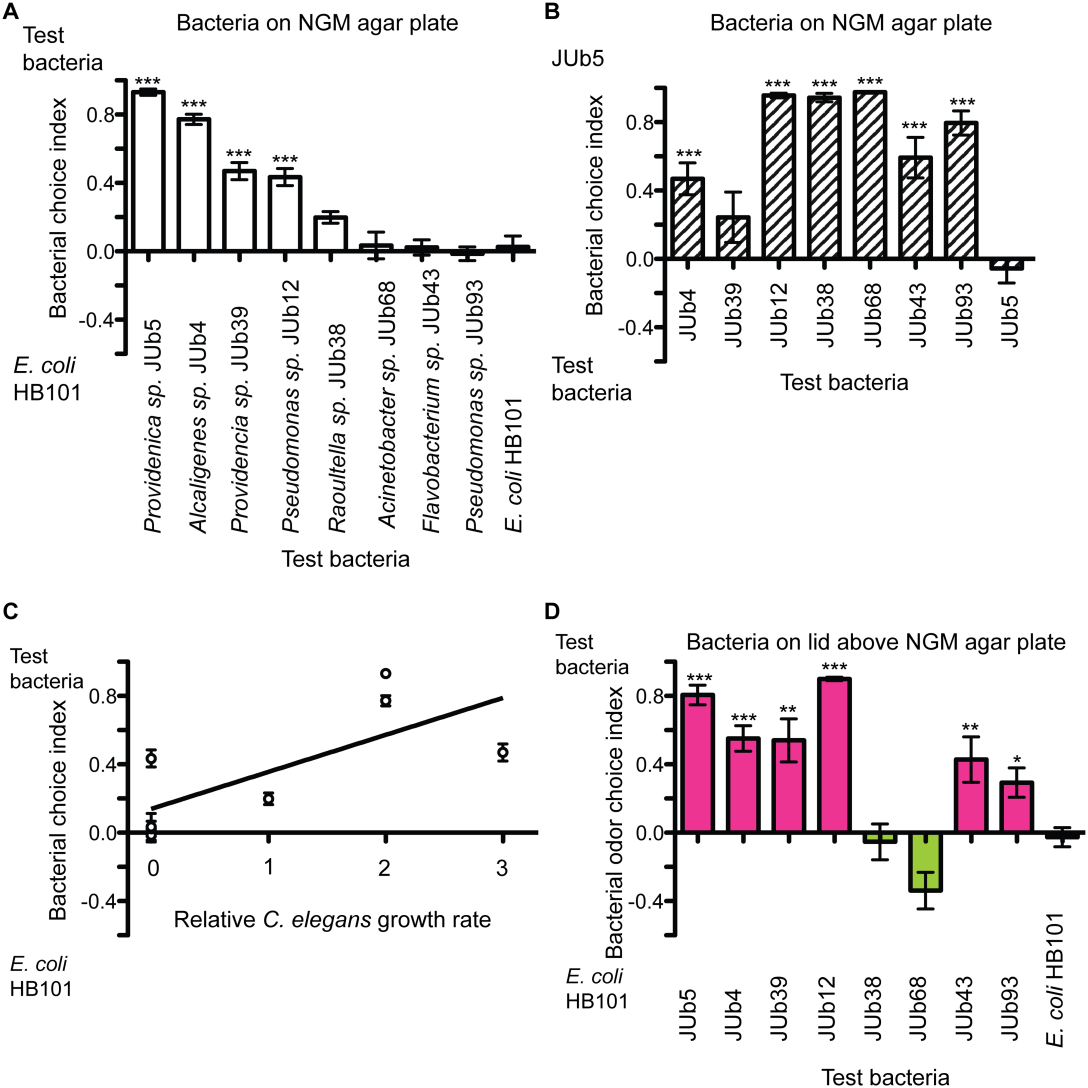
*C*. *elegans* preference for natural bacterial isolates. **(A)** Approximately 50–200 worms are placed on an agar plate between two patches of bacteria (OD600 = 10). Animals were allowed to move freely for 1 hour before plates were scored. Worms were given a choice between natural bacterial isolates and *E*. *coli* HB101. Bacterial choice index is number of worms on test bacteria minus number of worms on *E*. *coli* HB101 divided by the number of worms on test bacteria and number of worms on *E*. *coli* HB101. Wild-type (N2) showed a significant preference for four of the natural bacterial isolates. *** p < 0.001, ANOVA with Dunnett compared to choice index between two patches of *E*. *coli* HB101 bacteria (last bar), n ≥ 6 assays. **(B)** Worms were tested in bacterial choice between JUb5 and other natural bacterial isolates. Bacterial choice index is number of worms on JUb5 minus number of worms on test bacteria divided by the number of worms on JUb5 and number of worms on test bacteria. Wild-type (N2) showed a significant preference for JUb5 over six of the seven natural bacterial isolates tested. *** p < 0.001, ANOVA with Dunnett compared to choice index between two patches of JUb5 bacteria (last bar), n ≥ 6 assays. **(C)** Correlation between bacterial choice index (from panel A, natural bacterial isolate vs. *E*. *coli* HB101 bacterial patches on NGM agar plate) and relative growth of *C*. *elegans* on natural bacterial isolate compared to growth on *E*. *coli* OP50. Zero indicates same growth rate on natural bacterial isolate and *E*. *coli*; positive number indicates faster growth rate on natural bacterial isolate than *E*. *coli*. Growth data from previous study, Supplementary Dataset 3 [13]. A linear regression was calculated (r^2^ = 0.52). **(D)** Worms were tested for bacterial odor choice index, in which worms were placed on an agar plate equidistant from two patches of bacteria on lid of plate. Worms approach the area beneath the bacteria patches by olfactory chemotaxis. 1 μl of 1 M sodium azide NGM agar plate was placed on NGM agar plate directly below each bacterial patch to immobilize worms when they reached regions of plate below each bacterial patch. Bacterial odor choice index is number of worms under test bacteria minus number of worms under *E*. *coli* HB101 divided by the number of worms under test bacteria and number of worms under *E*. *coli* HB101. Worms showed a significant preference for six natural bacterial isolates over *E*. *coli* (pink) and did not show a significant preference for two natural bacterial isolates over *E*. *coli* (green). *** p < 0.001, ** p < 0.01, *p < 0.05, ANOVA with Dunnett compared to choice between two patches of *E*. *coli* HB101 bacteria on lid (last bar), n ≥ 6 assays. All behavioral assays conducted on at least two different days. Error bars represent SEM.

We evaluated the food preferences of *C*. *elegans* for eight different bacterial isolates found in the natural environment of *C*. *elegans*. The strains were isolated from three different habitats (compost, rotten apple, and slug) in two different geographic locations in France [13]. We used a bacterial choice assay in which worms are given a choice between the natural bacterial isolate and *E*. *coli* HB101, each on opposite sides of an agar plate [5]. In the one-hour bacterial choice assay, where bacterial patches are grown on NGM agar assay plates, bacterial preference is determined by attraction to volatile chemical cues as well as secreted cues released directly into the agar. In addition, in one hour, it is possible for worms to initially choose one bacterial patch and then leave that patch, which would reduce the preference score for that bacteria [6]. Thus, in the bacterial choice index, preference is determined by bacterial volatile cues and soluble cues as well as leaving rate of bacterial patches. In this assay, *C*. *elegans* showed a significant preference over *E*. *coli* HB101 for *Providencia sp*. JUb5, *Alcaligenes sp*. JUb4, *Providencia sp*. JUb39, and *Pseudomonas sp*. JUb12; a moderate non-significant preference for *Raoultella sp*. JUb38; and no preference for *Acinetobacter sp*. JUb68, *Flavobacterium sp*. JUb43, and *Pseudomonas sp*. JUb93 *(*Fig 1A).

We next examined preference in bacterial choices between natural bacterial isolates rather than between a natural isolate and *E*. *coli* HB101. These choices may be more similar to the bacterial choices *C*. *elegans* makes in the natural environment where *E*. *coli* is less likely to be present. In a series of choices between *Providencia sp*. JUb5 and each other bacterial isolate, *C*. *elegans* showed a strong preference for *Providencia sp*. JUb5 over six of the bacterial isolates (Fig 1B). *C*. *elegans* did not show a significant preference for JUb5 over JUb39, perhaps indicating that these two strains are perceived as equally attractive or similar to each other.

We then asked whether *C*. *elegans* exhibited food preference for bacterial isolates that were also beneficial food sources. Samuel *et al*., 2016 evaluated the growth rate of *C*. *elegans* on these bacterial isolates relative to the growth rate on *E*. *coli* OP50 [13]. We found a moderate correlation between bacterial preference (in the bacterial choice assay) and growth rate of *C*. *elegans* on bacteria (r^2^ = 0.52, Fig 1C). In general, the preferred species resulted in enhanced growth of *C*. *elegans* (JUb4, JUb5, and JUb39). *C*. *elegans* did not show preference for many of the species that did not enhance growth (JUb43, JUb68, and JUb93). One exception was that *C*. *elegans* showed a strong preference for *Pseudomonas sp*. JUb12, but JUb12 did not enhance growth. These results may suggest that *C*. *elegans* prefers bacteria that enhance growth rate, but many more isolates would need to be tested to support this conclusion.

### Olfactory preference behavior of *C*. *elegans* for natural bacteria

After determining the food preferences for *C*. *elegans* among the natural bacterial isolates, we next wanted to ascertain the olfactory preferences, based only on volatile chemical cues, of *C*. *elegans* for the natural bacterial isolates. To examine only olfactory preferences among bacterial strains, we used a different assay, the bacterial odor choice assay (adapted from Choi *et al*., 2016). In the bacterial odor choice assay, worm preference is only determined by volatile chemicals released by the bacteria and not by non-volatile cues or leaving rate. In this assay, squares of NGM agar containing bacterial patches were placed on the petri dish lid above an NGM agar plate with worms. Sodium azide was placed on the NGM agar directly below the bacterial patches on the lid in order to immobilize the worms when they reach the areas of the plate below the bacterial patches.

We found similar preferences for most of the bacterial isolates in the bacterial choice assay and the bacterial odor choice assay. In both assays, *C*. *elegans* showed a preference for four isolates (JUb4, JUb5, JUb12, and JUb39) and did not show a preference for two isolates (JUb38 and JUb68) over *E*. *coli* HB101 (Fig 1D). However, there were differences between the assays for two bacterial strains. In the bacterial odor choice assay, *C*. *elegans* showed a significant preference for two isolates (JUb43 and JUb93) but did not exhibit preference for them in the bacterial choice assay. Why would the preferences differ between the bacterial choice assay and bacterial odor choice assay? We hypothesize that JUb43 and JUb93 release repulsive non-volatile cues that negate the attractive volatile chemicals and so reduce preference for these bacteria in the bacterial choice assay. Another possibility is that during the one-hour bacterial choice assay, worms are initially attracted to JUb43 or JUb93, but then leave the bacterial patch, perhaps they perceive these bacterial strains be low food quality. Indeed, Samuel *et al*., 2016 evaluated the growth rate of *C*. *elegans* on different bacterial natural isolates and found that JUb43 and JUb93 did not enhance growth rate compared to growth on *E*. *coli* OP50 which may indicate that these strains provide poor nutrition. Thus, the degree of similarity in the results of these two assays seems to depend on the specific bacterial strains being assayed.

### Identification of volatiles released by natural bacteria

**Fig 2:**
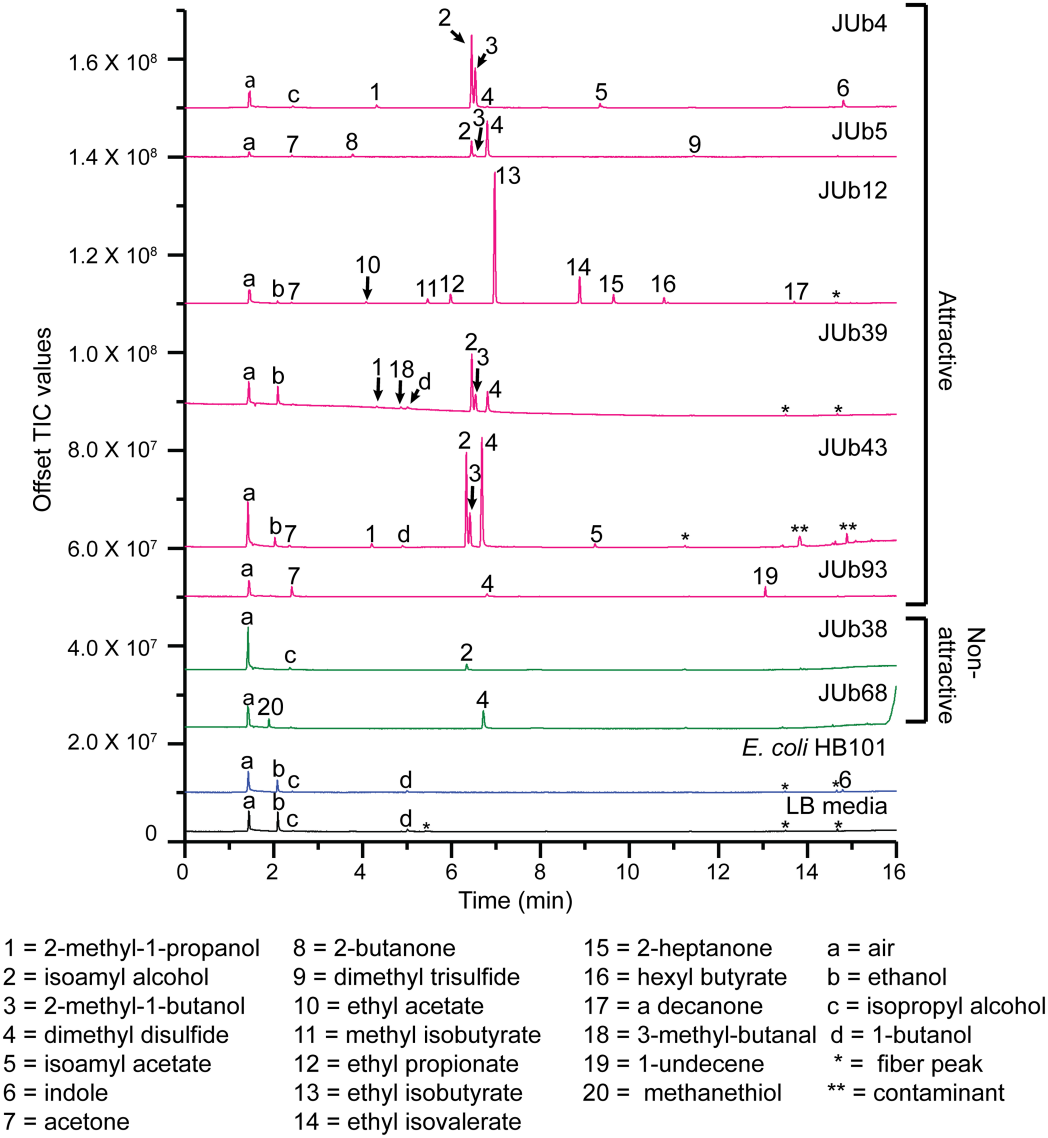
Gas chromatography-mass spectrometry of headspace of natural bacterial isolates, *E*. *coli* HB101 and LB media control. Overnight liquid cultures of bacteria were spotted on NGM agar plates (OD600 = 10) and incubated for 1 hour, then NGM agar squares with bacterial suspension were placed inside a GC-MS glass vial for five hours. A SPME fiber was inserted into the vial containing the bacteria to sample the volatile compounds in the headspace. Top, representative total ion chromatograms (TIC), from top to bottom: JUb4, JUb5, JUb12, JUb39, JUb43, JUb93, JUb38, JUb68, *E*. *coli* HB101and LB media control. Peaks present in bacterial samples, but not LB media, are numbered. Peaks present in LB media are labeled with letters. One asterisk indicates “fiber peaks,” volatile siloxanes released by the SPME fiber; two asterisks indicate contamination because also present in fiber blank control run prior to JUb43 sample analysis. Bottom, volatile organic compounds (VOCs) corresponding to labelled peaks. Peaks were identified tentatively with NIST 11 (National Institute of Standards and Technology) mass spectral library and confirmed with known standards. Retention times of VOCs in samples JUb43, JUb38, and JUb68 are shorter by approximately 0.1 minute compared to other samples analyzed because a small, contaminated segment of the column was removed from the inlet. See sample retention times in S1 Table. All bacterial samples were prepared and analyzed two or more times on different days.

We used solid-phase microextraction (SPME) gas chromatography-mass spectrometry (GC-MS) to identify the volatile chemicals present in the headspace of the eight natural bacterial isolates and *E*. *coli* HB101. The term headspace refers to the volume of air above the bacterial sample within the GC-MS glass vial. The bacteria were grown on agar plates in the same manner as grown for bacterial choice assays. Overnight liquid bacterial cultures were spotted on NGM agar (OD600 = 10) and incubated for one hour. Then NGM agar squares with bacterial suspension were placed inside a GC-MS vial for five hours and a SPME fiber was inserted into the vial to sample volatile organic compounds (VOCs) [10]. We identified the VOCs that were present in the headspace of the natural bacterial isolate, but not present in the headspace of *E*. *coli* HB101 or LB media spotted on NGM agar. We used the NIST 11 (National Institute of Standards and Technology) mass spectral library to identify tentatively the VOCs and confirmed the identity of the VOCs using standards made from pure chemicals (Fig 2).

**Fig 3:**
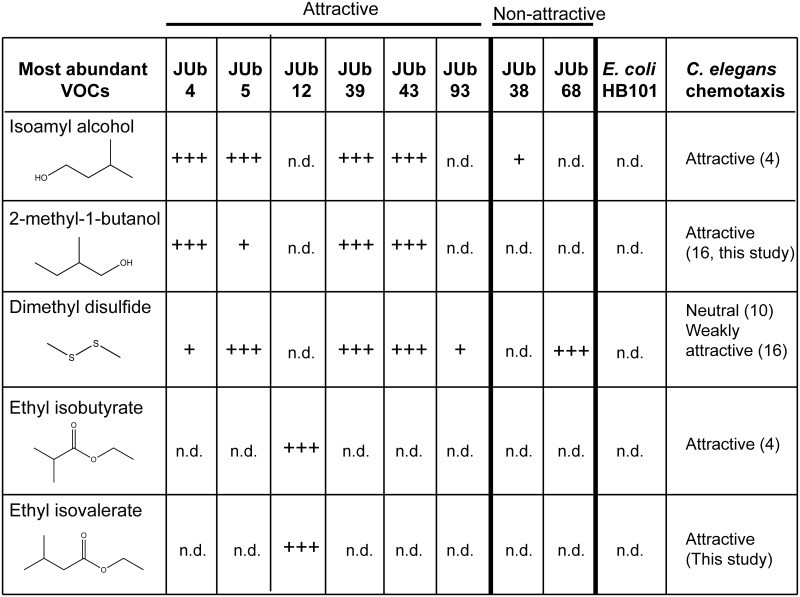
Summary of most abundant bacterial volatile organic compounds. VOCs were identified by SPME-GC-MS and confirmed with known standards. +++ indicates high abundance VOC with total ion chromatogram (TIC) peak area greater than 6.0 × 10^7^ counts; + indicates low abundance VOC with TIC peak area between 1.0 × 10^7^ and 6.0 × 10^7^counts; n.d. indicates not detected. Data from at least two different experimental replicates on different days. Attraction to *C*. *elegans* determined by bacterial odor choice assay (Fig 1D).

The headspace of each bacterial sample had a unique mixture of VOCs. We used chromatographic peak areas to define relatively high abundant VOCs (greater than 6.0 × 10^7^ counts) and low abundant VOCs (between 1.0 × 10^7^ and 6.0 × 10^7^ counts) (Fig 3). The headspace of five of the six attractive natural isolates (JUb4, JUb5, JUb12, JUb39, and JUb43) had two or three dominant compounds that were high abundance and several low abundant compounds (Fig 2). In comparison, the headspace of *E*. *coli* HB101 contained no high abundant VOCs and only had one low abundant VOC, indole, that was not in LB media (Fig 2). This result was in contrast to other studies of *E*. *coli* which identified several VOCs in *E*. *coli* headspace including indole [19]. This difference is likely because the experimental growth conditions in this study slow the growth and metabolism of *E*. *coli*. In this study, *E*. *coli* was grown at 20 °C on NGM agar plates while *E*. *coli* has been typically grown at 37 °C in liquid LB in most *E*. *coli* VOC studies [19]. *E*. *coli* is cultured at 20 °C for our VOC analysis so that the bacterial state will be similar to the bacteria in choice assays conducted at 20 °C, an optimal temperature for *C*. *elegans*. However, while 20 °C likely slows down the growth and metabolism of *E*. *coli* resulting in the release of fewer volatiles, 20 °C may be close to the optimal temperature for growth of the natural environmental isolates which may explain why they release more abundant VOCs.

**Fig 4:**
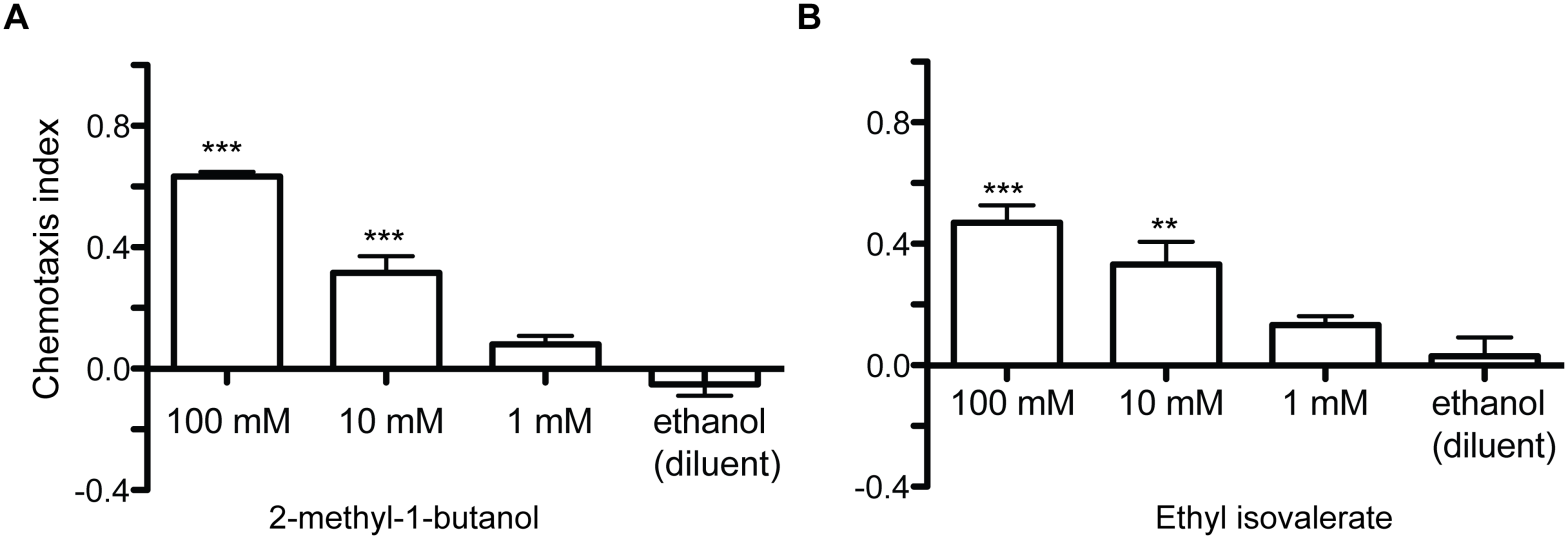
Chemotaxis to volatile organic compounds released by natural bacterial isolates. All volatile organic compounds were diluted in ethanol. (**A**) 2-methyl-1-butanol (**B)** Ethyl isovalerate. *** p < 0.001, ** p < 0.01 ANOVA with Dunnett compared to chemotaxis between ethanol (the diluent) on both sides of the chemotaxis plate (last bar), n ≥ 6 assays. Assays conducted on at least two different days. Error bars represent SEM.

Interestingly, isoamyl alcohol was one of the most abundant VOCs in four attractive bacterial isolates (JUb4, JUb5, JUb39 and JUb43). Isoamyl alcohol is a well-known attractant over a range of concentrations from neat to 10^−4^ dilution and has been used in many studies of *C*. *elegans* [4,20,21]. However, to our knowledge, this is the first time that isoamyl alcohol has been shown to be released by a bacterial strain to which *C*. *elegans* is attracted. The other abundant VOCs in the headspace of these bacteria were 2-methyl-1-butanol (JUb4, JUB39 and JUb43) and dimethyl disulfide (JUb5, JUb39, and JUb43). The odorant 2-methyl-1-butanol was found to be attractive to *C*. *elegans* in chemotaxis assays at a range of concentrations [16](Fig 4A). Dimethyl disulfide has been previously shown to be weakly attractive or neutral at a range of concentrations [10,16]. In addition, several of the low abundant odorants present in the headspace of these strains have also been shown to be attractive to *C*. *elegans*: including 2-methyl-1-propanol, acetone, 2-butanone, isoamyl acetate and ethyl acetate [4]. These odorants may contribute to the attractiveness of the natural bacterial odor as well. The attractive isolate JUb12 had a very different odor profile: the two most abundant VOCs released by JUb12 were ethyl isobutyrate and ethyl isovalerate. Ethyl isobutyrate has previously been shown to be attractive [4]. We found that ethyl isovalerate was attractive at a range of concentrations in *C*. *elegans* chemotaxis assays (Fig 4B). Both ethyl isobutyrate and ethyl isovalerate are described as “fruity” odors [22].

The odor of the JUb93 strain was found to be moderately attractive, but JUb93 headspace did not contain any high abundant volatile chemicals. However, JUb93 headspace did contain acetone which has been shown to be attractive to *C*. *elegans* at a range of concentrations from 1 mM to 1 M [4,10]. Thus, acetone alone or in combination with the other low abundant volatile chemicals in the headspace of JUb93 may be the basis of its moderate attractiveness to *C*. *elegans*.

The two non-attractive bacterial isolates, JUb38 and JUb68, did not contain any high abundant volatile chemicals. The chromatogram of the JUb38 headspace contained a very small peak representing isoamyl alcohol. We calculated the approximate concentrations of isoamyl alcohol (IAA) in the headspace of the different bacterial isolates based on the chromatographic peak areas relative to peak area of IAA standard of known concentration. We found that of the samples that contained isoamyl alcohol, JUb38 contained the least IAA. The bacterial headspaces contained (in order from most to least IAA): JUb43, ~45 mM IAA; JUb4, ~20 mM IAA; JUb39, ~20 mM IAA; JUb5, ~6 mM IAA; and JUb38, ~1 mM IAA. JUb68 headspace did not have any detectable isoamyl alcohol. The most abundant volatile chemical in JUb68 was dimethyl disulfide which has been previously shown to be weakly attractive or neutral at a range of concentrations [10,16].

Taken together, four attractive bacterial isolates released a relatively high amount of isoamyl alcohol (JUb4, JUb5, JUb39and JUb43) while non-attractive isolates released a very low amount (JUb38) or non-detectable amount (JUb68). Two attractive bacterial isolates (JUb12 and JUb93) did not release isoamyl alcohol, but did release other odorants which are attractive to *C*. *elegans*. Thus, isoamyl alcohol is likely an important attractive volatile for some bacterial isolates in the natural environment of *C*. *elegans*.

## Discussion

We determined the food preferences of *C*. *elegans* for different bacteria found in their natural environment, determined their olfactory preferences for odors of these bacteria based only on volatile cues, and identified some attractive bacterial odorants. We found that *C*. *elegans* preferred the odor of six natural bacterial isolates over *E*. *coli* HB101 and that four of these isolates released isoamyl alcohol.

### Bacterial food preference behavior

We used two assays to measure bacterial preference. In the bacterial choice assay, the bacterial preference of *C*. *elegans* was determined based on volatile cues, soluble cues, and rate of leaving bacterial patches on the assay plate. In the bacterial odor choice assay, the bacterial preference of *C*. *elegans* was determined only by volatile chemicals released by the bacterial isolate because the bacterial patches were presented on the lid of the assay plate. For most of the bacterial isolates tested in this study, the preference results were similar for both assays. The two bacterial isolates which yielded different results were JUb43 and JUb93. Both strains were attractive in the bacterial odor choice assay, but were not attractive in the bacterial choice assay. This result likely indicates that JUb43 and JUb93 release attractive volatile chemicals, but that these strains also release repulsive soluble cues, or induce a high rate of leaving, or a combination of both. The other six bacterial isolates were similarly attractive in both assays, likely indicating that for these bacteria, volatile cues were the largest contributor to preference in the bacterial choice assay. Thus, the degree volatile cues determine food preference depends on the specific bacterial strain being examined.

Although *C*. *elegans* has been shown to exhibit innate preferences for different species of bacteria, to our knowledge, this is the first time preferred bacteria isolated from the natural habitat of *C*. *elegans* have been identified [5,6]. We found that *C*. *elegans* preferred bacterial isolates from the genera *Providencia*, *Alcaligenes* and *Pseudomonas*. Have these genera of bacteria previousl*y* been shown to be preferred by *C*. *elegans* in other food choice studies? To our knowledge, *Providencia* and *Alcaligenes* strains have not been examined before. However, a *Pseudomonas* isolate (from soil and not co-located with *C*. *elegans*) was shown to be preferred in a food choice assay conducted over 48 hours based on both olfactory and non-olfactory cues [23]. Yu *et al*., 2015 suggested that *C*. *elegans* preferred this bacterium because of its rapid growth and high respiration as indicated by emitted carbon dioxide. The study also showed that *C*. *elegans* that fed on these more active bacteria had increased numbers of progeny [23].

In addition to olfactory cues and bacterial growth rate, another criterion for food preference is bacterial cell size. *C*. *elegans* has been shown to prefer bacteria with smaller cell size which are easier to eat and promote faster growth of *C*. *elegans* [24]. Two *Pseudomonas* isolates were shown to have small size and promote faster development of *C*. *elegans*, but they were not tested for food preference [24].

Although these studies may suggest *Pseudomonas* is a generally preferred bacterial food, in this study, the preference behavior towards two different *Pseudomonas* isolates differed. One isolate of *Pseudomonas* (JUb12) was preferred in both bacterial preference assays and one (JUb93) was only weakly preferred in the bacterial odor choice assay. This difference in preference for *Pseudomonas* bacterial isolates is not surprising because the *Pseudomonas* genus is large and complex with over 100 species and much intra-species variation [25]. Consistent with this, Samuel *et al*., 2016 found a wide range in effects of different natural *Pseudomonas* isolates on *C*. *elegans* growth and physiology [13]. Therefore, food preference will be need to be determined for more bacterial isolates in order to determine which, if any, genera are consistently attractive as well as the full set of criteria that govern food choice in *C*. *elegans*.

A moderate correlation between the preferred bacterial strain and bacterial strain that enhanced the rate of growth of *C*. *elegans* from L1 to adult may suggest that *C*. *elegans* has evolved to prefer beneficial bacteria. Moreover, one preferred species, *Providencia sp*. JUb39, was shown to be very beneficial because it was protective against the negative effects of other detrimental bacteria on *C*. *elegans* growth [13]. However, more isolates would need to be tested in order to support the connection between preference and beneficial bacteria.

### Volatile chemicals that attract *C*. *elegans*

Although it has long been known that VOCs that are byproducts of bacterial metabolism attract *C*. *elegans*, it has not been known which of these VOCs are relevant to *C*. *elegans* in its natural environment. We found that isoamyl alcohol, a very well-studied *C*. *elegans* attractant, was one of the most abundant odors released by four attractive species of bacteria. To our knowledge, this is the first time that isoamyl alcohol has been shown to be released by an attractive microbe. However, other VOCs important for attraction to several pathogens have been identified: acetone and 2-butanone for S. *marcescens*; 2-heptanone for *B*. *nematocida*; and acylated homoserine lactone autoinducer for *P*. *aeruginosa* [7,8,10]. Another detrimental microbe, nematophagous fungus, *Arthrobotrys oligospora*, which preys on *C*. *elegans* has also been shown to release several attractive odorants, including methyl 3-methyl-2-butenoate (MMB) [16].

In addition, we found that preference for a VOC does not necessarily correlate with the abundance of that VOC. For example, dimethyl disulfide was one of the most abundant odors in two of the isolates (JUb5 and JUb39), but was only a weak or neutral attractant in *C*. *elegans* chemotaxis assays [10,16]. Moreover, worms are known to be attracted to the some of the lower abundant volatiles, such as 2-butanone (JUb5), acetone (JUb5, JUb12, and JUb93) and 2-heptanone (JUb12) and these volatiles likely also contribute to the attractiveness of bacterial odor blends [4].

## Conclusion

We determined that isoamyl alcohol, a well-studied *C*. *elegans* attractant, is likely to be an ethologically relevant odor for *C*. *elegans* because it is released by four attractive natural bacterial isolates.

## Supporting information

S1 Table Retention times of identified volatile organic compounds (VOCs) in bacterial headspace. (PDF)
