# Supplementary material for: Identification of attractive odorants released by preferred bacterial food found in the natural habitats of C. elegans
Source: PLoS One. 2018 Jul 23;13(7):e0201158. doi: 10.1371/journal.pone.0201158 (PMC6056031; doi:10.1371/journal.pone.0201158)
Supplement: S1 Table — (PDF) [file pone.0201158.s001.pdf]

Supplemental Table 1. Retention times of identified volatile organic compounds (VOCs) in bacterial headspace. +++ indicates high abundant VOC with total ion chromatogram (TIC) peak greater than 6.0 x 10^7 counts; + indicates low abundant VOC with TIC peak area between 1.0 x 10^7 and 6.0 x 10^7counts. Data from at least two different experimental replicates on different days.

			Attractive						Non-attractive			
**Volatile Organic Compounds**	**Reten-tion time (min)**	**Label in Fig. 2**	**JUb 4**	**JUb 5**	**JUb 12**	**JUb 39**	**JUb 43**	**JUb 93**	**JUb 38**	**JUb 68**	***E. coli***	**LB**
Methanethiol	2.01	20								+		
Acetone	2.48	7		+	+		+	+				
Isopropyl alcohol	2.51	c	+						+		+	+
2-Butanone	3.84	8		+								
Ethyl acetate	4.14	10			+							
2-Methyl-1-propanol	4.38	1	+			+	+					
3-Methyl-butanal	4.92	18				+						
1-Butanol	5.07	d				+	+				+	+
Methyl isobutyrate	5.51	11			+							
Ethyl propionate	6.03	12			+							
Isoamyl alcohol	6.50	2	+++	+++		+++	+++		+			
2-Methyl-1-butanol	6.58	3	+++	+		+++	+++					
Dimethyl disulfide	6.85	4	+	+++		+++	+++	+		+++		
Ethyl isobutyrate	7.01	13			+++							
Ethyl isovalerate	8.92	14			+++							
Isoamyl acetate	9.38	5	+				+					
2-Heptanone	9.68	15			+							
Hexyl butyrate	10.80	16			+							
Dimethyl trisulfide	11.48	9		+								
1-undecene	13.08	19						+				
A decanone	13.73	17			+							
Indole	14.82	6	+								+	
